# Impact of Specialized Pro-Resolving Lipid Mediators on Craniofacial and Alveolar Bone Regeneration: Scoping Review

**DOI:** 10.1590/0103-6440202406133

**Published:** 2024-10-25

**Authors:** Emanuel da Silva Rovai, Mackeler Polassi, Marcela Iunes da Silveira, Sandy Lima Araújo, Thomas Van Dyke, Nidia C. Castro dos Santos

**Affiliations:** 1 Department of Diagnosis and Surgery, Institute of Science and Technology, São Paulo State University, São José dos Campos, SP, Brazil; 2 Dental Research Division, Guarulhos University, Guarulhos, SP, Brazil; 3 Albert Einstein School of Dental Medicine, Albert Einstein Israelite Hospital, São Paulo, SP, Brazil; 4 The ADA Forsyth Institute, Cambridge, MA, United States; 5 Harvard School of Dental Medicine, Harvard University, Boston, MA, United States

**Keywords:** Bone regeneration, lipoxins, maresins, resolvins, specialized pro-resolving lipid mediators

## Abstract

Craniofacial bone defects caused by tumors, trauma, long-term tooth loss, or periodontal disease are a major challenge in the field of tissue engineering. In periodontitis and peri-implantitis, reconstructive therapy is also a major challenge for the dental surgeon. Lipoxins, resolvins, protectins, and maresins, known as specialized pro-resolving lipid mediators (SPMs), have been widely studied in the field of dental, oral, and craniofacial research for bone regeneration for their actions in restoring tissue homeostasis and promoting tissue healing and regeneration. Therefore, this study focuses on a survey of the use of SPMs for craniofacial and alveolar bone regeneration. Thus, electronic searches of five databases were performed to identify pre-clinical studies that evaluated the actions of SMPs on craniofacial and alveolar bone regeneration. Of the 523 articles retrieved from the electronic databases, 19 were included in the analysis. Resolvin (Rv) E1 was the mostly assessed SPM (n=8), followed by maresins (Ma) R1 (n=3), lipoxins (Lx) A4 (n=3), RvD1 (n=3), RvD2 (n=1), LxB4 (n=1), and maresin (M)-CTR3 (n=1). Meta-analysis showed that SPMs increased the newly formed bone by 14.85% compared to the control group (p<0.00001), decreased the area of the remaining defect by 0.35 mm2 (p<0.00001), and decreased the linear distance between the defect to the bone crest by 0.53 mm (p<0.00001). RvE1 reduced inflammatory bone resorption in periodontal defects and calvarial osteolysis and enhanced bone regeneration when RvE1 was combined with a bovine bone graft. RvD2 induced active resolution of inflammation and tissue regeneration in periapical lesions, while RvD1 controlled the inflammatory microenvironment in calvarial defects in rats, promoting bone healing and angiogenesis. MaR1 induced the proliferation and migration of mesenchymal stem cells, osteogenesis, and angiogenesis in calvarial defects, and benzo (b)-LxA4 and LxA4 promoted bone regeneration calvarial and alveolar bone defects in rats, inducing regeneration under inflammatory conditions. In summary, SPMs have emerged as pivotal contributors to the resolution of inflammation and the facilitation of bone neoformation within craniofacial and alveolar bone defects. These results are based on pre-clinical studies, in vivo and in vitro, and provide an updated review regarding the impact of SPMs in tissue engineering.

## Resumo

Defeitos ósseos craniofaciais causados por tumores, traumas, perda dentária prolongada ou doenças periodontais representam um grande desafio no campo da engenharia tecidual. Em casos de periodontite e peri-implantite, a terapia reconstrutiva também representa um grande desafio para o cirurgião-dentista. Lipoxinas, resolvinas, protectinas e maresinas, conhecidas como mediadores lipídicos especializados pró-resolutivos (SPMs - Specialized Pro-resolving Lipid Mediators), têm sido amplamente estudadas no campo da pesquisa odontológica para a regeneração óssea devido às suas ações na restauração da homeostase tecidual e na promoção da cicatrização e regeneração tecidual. Portanto, este estudo foca em uma revisão sobre o uso dos SPMs para a regeneração óssea craniofacial e alveolar. Assim, foram realizadas buscas eletrônicas em cinco bases de dados para identificar estudos pré-clínicos que avaliaram as ações dos SPMs na regeneração óssea craniofacial e alveolar. Dos 523 artigos recuperados das bases de dados eletrônicas, 19 foram incluídos na análise. A resolvina (Rv) E1 foi o SPM mais avaliado (n=8), seguida pelas maresinas (Ma) R1 (n=3), lipoxinas (Lx) A4 (n=3), RvD1 (n=3), RvD2 (n=1), LxB4 (n=1) e maresina (M)-CTR3 (n=1). A meta-análise mostrou que os SPMs aumentaram o osso recém-formado em 14,85% em comparação ao grupo controle (p<0,00001), diminuíram a área do defeito remanescente em 0,35 mm² (p<0,00001) e reduziram a distância linear entre o defeito e a crista óssea em 0,53 mm (p<0,00001). A RvE1 reduziu a reabsorção óssea inflamatória em defeitos periodontais e osteólise em calvárias e aumentou a regeneração óssea quando combinada com um enxerto ósseo bovino. A RvD2 induziu a resolução ativa da inflamação e a regeneração tecidual em lesões periapicais, enquanto a RvD1 controlou o microambiente inflamatório em defeitos em calvárias em ratos, promovendo a cicatrização óssea e a angiogênese. A MaR1 induziu a proliferação e migração de células-tronco mesenquimais, osteogênese e angiogênese em defeitos em calvárias, e a benzo (b)-LxA4 e a LxA4 promoveram a regeneração óssea em defeitos craniofaciais e alveolares em ratos, induzindo a regeneração sob condições inflamatórias. Em resumo, os SPMs emergiram como contribuintes fundamentais para a resolução da inflamação e a facilitação da neoformação óssea em defeitos craniofaciais e alveolares. Esses resultados são baseados em estudos pré-clínicos, in vivo e in vitro, e fornecem uma revisão atualizada sobre o impacto dos SPMs na engenharia tecidual.

**Figure:**
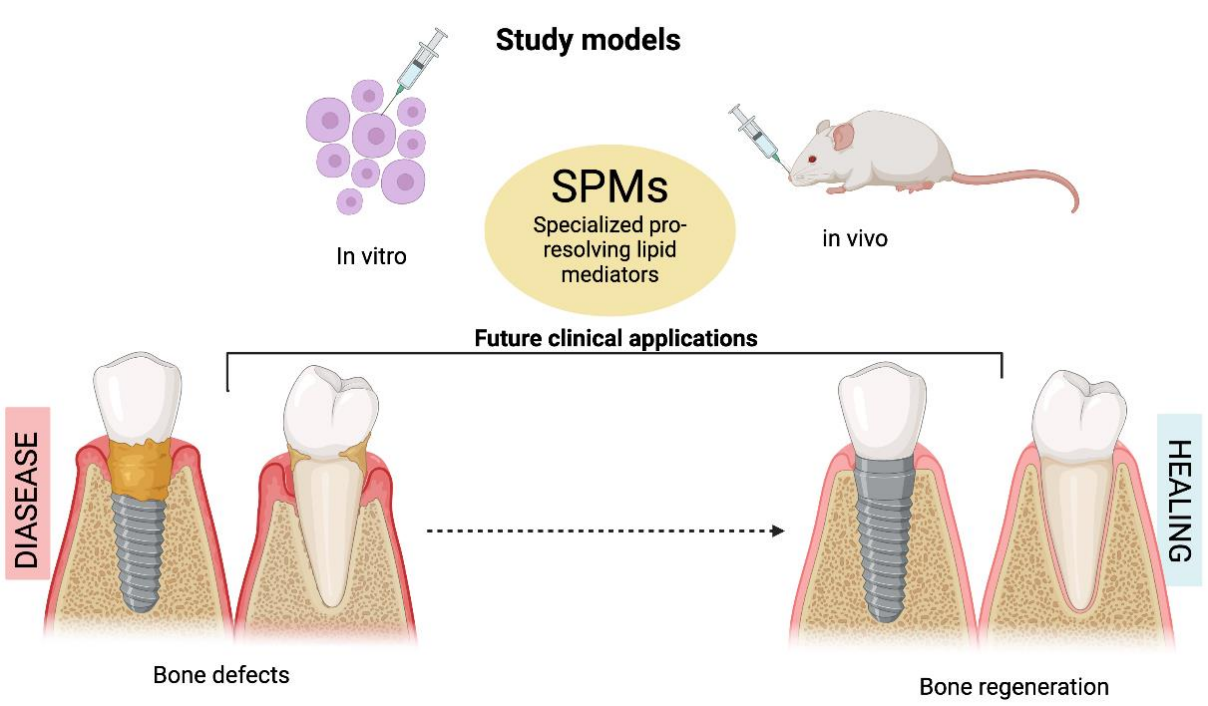

## Introduction

Craniofacial bone defects resulting from tumors, trauma, long-term tooth loss, or periodontal disease pose significant challenges in tissue engineering. Various types of grafts have been developed over the years in an attempt to meet this demand. Given their limitations, such as the need for a donor area in the case of autogenous grafts, or the lack of osteogenic cells in the case of allografts and xenografts, other alternatives have shown promise, such as the use of scaffold materials ^1^.

When it comes to periodontitis and peri-implantitis, reconstructive therapy is also a major challenge for the dental surgeon. These are inflammatory diseases characterized by the destruction of the support apparatus around teeth and implants, respectively, with a histopathological difference between the two, which justifies the different levels of progression between them ^2^. When not treated, inflammation leads to irreversible loss of these tooth and implant insertion tissues with concomitant alveolar bone loss. Its etiopathogenesis is complex and is due to a dysbiosis of the periodontal/peri-implant microbiota. Although it is much debated whether this dysbiosis is caused by periodontal disease or whether periodontal disease causes it, a bidirectional interrelationship is very likely ^2^.

As these are inflammatory diseases, understanding inflammatory physiology has been widely studied in the search for therapies. Acute inflammation is the body's initial response to a pathogenic aggression to eradicate it and, if not resolved, there is a progression to a chronic inflammatory condition ^3^. To resolve inflammation, there is an active exchange between mediators such as leukotrienes and prostaglandins, which will endogenously produce pro-resolution lipid mediators from arachidonic acid and essential fatty acids such as omega-3 docosahexaenoic acid and eicosapentaenoic acid ^4^. Lipoxins, resolvins, protectins, and maresins together form a new genus of pro-resolution mediators called specialized pro-resolving lipid mediators (SPMs). These act in host defense, tissue remodeling, pain, and organ protection ^5^. The restorative mechanism provided by SPMs is by inhibiting leukocyte infiltration and the generation of cytokines and chemokines, as well as inducing polymorphonuclear (PMN) apoptosis and increasing macrophage clearance of apoptotic cells ^1^ ^,^ ^5^.

Studies show that patients with periodontitis have elevated levels of pro-inflammatory mediators including cytokines, chemokines, and metalloproteinases in the gingival crevicular fluid (GCF) due to increased neutrophil recruitment ^4^. There is also an increase in early response lipid mediators such as prostaglandin E2 (PGE2) and leukotriene B4 (LTB4), as well as an up-regulation of COX2 ^6^. There are reports of the role of resolvin RvD1 in controlling inflammation, promoting bone healing, and acting on angiogenesis in critical bone defects in rat calvaria ^1^. In this context, SPMs have been widely studied in the field of dental, oral, and craniofacial research a promising therapy since they act on the inflammation and recovery of affected tissues, restoring their homeostasis ^3^.

Multiple in vitro and in vivo studies used SPMs in craniofacial and alveolar bone regeneration using different mediator and study models, presenting various outcomes. Therefore, this systematic review focuses on a survey of the use of SPMs for craniofacial and alveolar bone regeneration. The hypothesis is that SPMs can improve craniofacial and alveolar bone regeneration.

## Materials and methods

This review followed a systematic search for studies encompassing the use of SPMs for craniofacial and alveolar bone regeneration. The search utilized the following terms: ((eicosapentaenoic acid or omega-3 or omega 3 or docosahexaenoic or ω-3 or resolvin or maresin or poly-unsaturated fatty acids or lipoxins or protectin) AND (Bone or osseous or osteoblast or osteocyte or osteoclast or mesenchymal stem cells or periodontal disease or periodontitis)) AND (regeneration or healing). Search strategies were developed at Pubmed/MEDLINE, Scopus, LILACS, and EMBASE databases up to December 2023.

The primary aim of this systematic review was to investigate the impact of SPMs on the regeneration and repair of craniofacial and periodontal bone defects. Specifically, the research question guiding this inquiry was formulated as follows: What are the actions of SPMs on bone or periodontal regeneration?

The selection of studies for inclusion in this review was guided by a predefined set of inclusion and exclusion criteria, structured according to the PICO (Population, Intervention, Comparison, Outcomes) framework:

Population (P): The targeted population comprised individuals with bone defects necessitating regeneration on craniofacial defects and/or periodontal.

Intervention (I): The intervention of interest centered on the administration of SPMs.

Comparison (C): Comparative analyses were conducted concerning the outcomes observed in the absence of SPMs.

Outcomes (O): The primary outcomes of interest encompass craniofacial and alveolar bone regeneration.

Studies in vitro and in vivo (animal studies) were considered for this study.

The Rayyan application was employed for article selection.

### Inclusion and Exclusion Criteria

The first step involved the preliminary analysis of titles that could meet the inclusion criteria, aiming to identify potentially relevant articles. Before this process, all items found through electronic searches were grouped into a single list, with duplicates excluded using the Rayyan.ia software (https://new.rayyan.ai/reviews/925120/overview). The keywords used in the search were duly identified (NCCS and ESR).

Subsequently, two evaluators, (MP and MI), independently conducted the analysis of abstracts (when available) of all reports identified in Rayyan's unique list. Access to the full article was made when studies met the inclusion criteria or when the summarized data were insufficient to evaluate them.

The eligibility of full articles identified as potentially relevant in the initial screening phase was then verified, exemplifying methodological stringency, two independent reviewers (MP and MI), conducted manual data analysis utilizing the Rayyan platform and the inter-examiner agreement between the two evaluators was 91%. Only original studies published in English and directly addressing the predefined PICO question were considered for inclusion. Articles falling outside the scope of the review, such as commentaries, editorials, hypotheses, and research unrelated to the designated outcomes, were excluded based on a meticulous assessment of titles and abstracts. To ensure comprehensive coverage, the included articles were categorized into two distinct groups: in vivo studies and in vitro studies.

While the Rayyan application facilitated the initial stages of article screening and selection, the inherent complexity and subjectivity of the systematic review process necessitated the intervention of a third reviewer (NCCS), to resolve discrepancies and ensure methodological robustness. This intervention was particularly critical in addressing the 74 articles that initially presented disagreement, underscoring the importance of expert oversight in navigating intricate review processes.

### Data Extraction

Data extraction procedures were executed to extract relevant information about the use of SPMs in bone regeneration.

A crafted data extraction form was employed to record the information from each included study, encompassing elements such as article title, publication date, authors, number of participants, study nature (in vivo or in vitro), administered dosage, demographic characteristics of the sample, follow-up period, bias risk assessment (high, medium, and low), assessment points, and outcomes obtained. A collaborative analysis between reviewers (MP and MI) determined the necessity for full reading and data extraction, conducted independently. In case of disagreement between the reviewers, a consensus was reached through discussion and joint deliberation.

Only studies explicitly employing these mediators were considered for inclusion, with data retrieved from the original texts or figures. When raw data were unavailable, results were transformed into relative proportions to facilitate comparative analyses.

## Results

**Figure 1:**
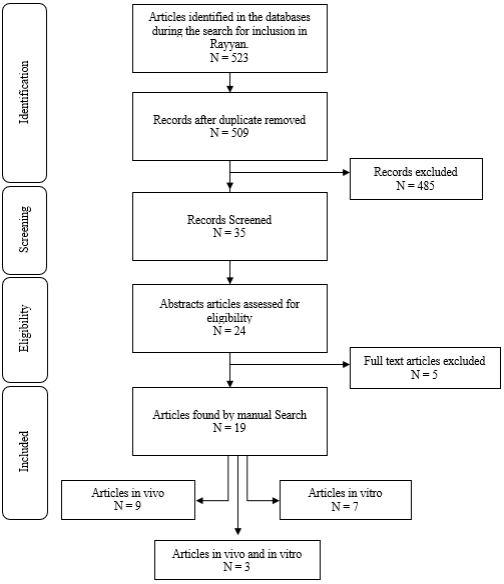
Study flowchart

In this study, 523 articles published between 1999 and 2023 were identified, thoroughly examined, and selected according to the inclusion criteria. Using the Rayyan software, 14 duplicates were detected and removed. Additionally, 35 articles were selected for title analysis, resulting in exclusion based on the established criteria. Out of the total, 24 were read in full, and 18 articles were selected for inclusion in this systematic review. An additional study was included after a manual search in the literature. This resulted in a total of 19 articles for reading and data extraction. The search in the grey literature did not yield new studies. The flowchart of the search and selection process, along with the reasons for excluding potential studies, is presented in Figure 1.

Resolvin (Rv) E1 was the mostly assessed SPM (n=8) ^7^ ^,^ ^8^ ^,^ ^9^ ^,^ ^10^ ^,^ ^11^ ^,^ ^12^ ^,^ ^13^ ^,^ ^14^, followed by maresins (Ma) R1 (n=3) ^11^ ^,^ ^14^ ^,^ ^15^, lipoxins (Lx) A4 (n=3) ^5^ ^,^ ^16^ ^,^ ^18^, RvD1 (n=3) ^1^ ^,^ ^20^ ^,^ ^21^, RvD2 (n=1) ^19^, LxB4 (n=1) ^17^, and maresin (M)-CTR3 (n=1) ^22^.

**Figure 2:**
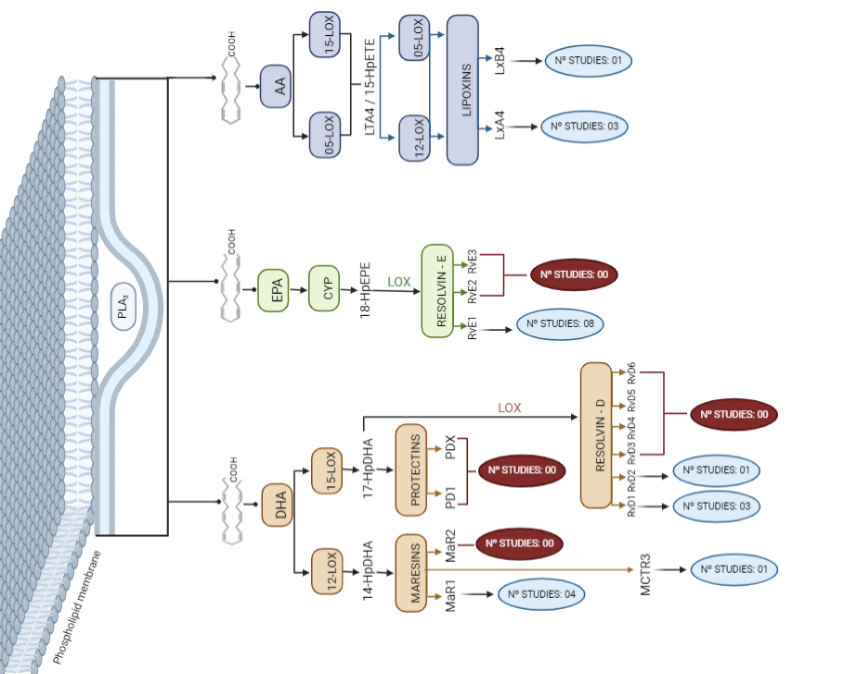
Quantification of studies by SPMs classification according to its biological formation.

Figure 2 presents the results of the quantitative analysis according to the findings in the in vivo and in vitro studies, revealing that regardless of the type of study, there are no records of the use of protectins, as well as resolvins E2, E3, D3, D4, D5, and D6, and maresins R2.

Overall, studies with resolvins comprise twelve articles ^1^ ^,^ ^7^ ^,^ ^8^ ^,^ ^9^ ^,^ ^10^ ^,^ ^11^ ^,^ ^12^ ^,^ ^13^ ^,^ ^14^ ^,^ ^19^ ^,^ ^20^ ^,^ ^21^; maresins comprise five articles ^11^ ^,^ ^12^ ^,^ ^13^ ^,^ ^15^ ^,^ ^22^; lipoxins comprise four articles ^5^ ^,^ ^16^ ^,^ ^17^ ^,^ ^18^; and there were no studies regarding the actions of protectins.

Box 1 presents the characteristics of the animal model studies. Collectively, in vivo studies revealed promising results regarding the potential of SPMs in bone regeneration across various animal models and bone and periodontal defect conditions.

Studies assessing RvE1 ^9^ ^,^ ^10^ have demonstrated that this SMP reduces inflammatory bone resorption in models of alveolar bone defects and calvarial osteolysis. Both studies employed histological and immunohistochemical evaluation methods to determine the effects of RvE1 on bone regeneration. One study investigated the role of RvE1 in calvarial defects and observed enhanced bone regeneration when RvE1 was combined with a bovine bone graft ^13^.

Other SPMs, such as RvD2, RvD1, and MaR1, have shown potential in bone regeneration in animal models. RvD2 induces active resolution of inflammation and tissue regeneration in periapical lesions ^19^. Similarly, research on the effect of RvD1 in calvarial defects in rats controlled the inflammatory microenvironment, promoting bone healing and angiogenesis ^1^. Evaluation of MaR1 in calvarial defects promoted the proliferation and migration of mesenchymal stem cells, osteogenesis, and angiogenesis ^15^

Studies have also explored the potential of other SPMs, such as benzo (b)-LxA4 and LxA4, respectively, in bone regeneration using models of periodontal and calvarial defects in rats. Both studies highlighted the improvement of bone regeneration under inflammatory conditions ^16^ ^,^ ^18^.

Box 2presents the characteristics of the in vitro studies. The reviewed in vitro studies investigated the effects of SPMs on bone regeneration and inflammation resolution.

The main cell assay employed and identified was the Enzyme-linked immunosorbent assay (ELISA) ^5^ ^,^ ^9^ ^,^ ^11^ ^,^ ^14^ ^,^ ^20^ ^,^ ^22^, used to quantify the expression of pro-inflammatory cytokines (IL-1β and TNF-α), demonstrating the effects of SPMs on modulating the inflammatory response. Real-time polymerase chain reaction (PCR) was applied to analyze the gene expression of markers related to cell differentiation and tissue regeneration ^9^ ^,^ ^15^ ^,^ ^17^ ^,^ ^21^.

Additionally, alizarin red staining was used to evaluate bone mineralization, indicating the formation of mineralized matrix ^15^ ^,^ ^17^ ^,^ ^18^, as well as alkaline phosphatase (ALP) assays to assess osteoblastic activity, indicating cell differentiation towards bone formation ^9^ ^,^ ^11^ ^,^ ^18^. Lastly, western blotting was employed to analyze the expression of proteins involved in cell differentiation and inflammation regulation ^8^ ^,^ ^12^ ^,^ ^17^.

Other significant findings from the in vitro studies for potential therapeutic applications revealed the effects of LxB4, MCTR3, and MaR1 on periodontal ligament stem cells, highlighting the ability of these mediators to modulate cellular function and create a favorable microenvironment for tissue regeneration ^15^ ^,^ ^17^ ^,^ ^22^, as well as RvD1 and LxA4 on periodontal ligament cells, evidenced their pro-resolution properties and their ability to promote proliferation, migration, and osteogenic differentiation of these cells ^18^ ^,^ ^20^.

**Box 1:**
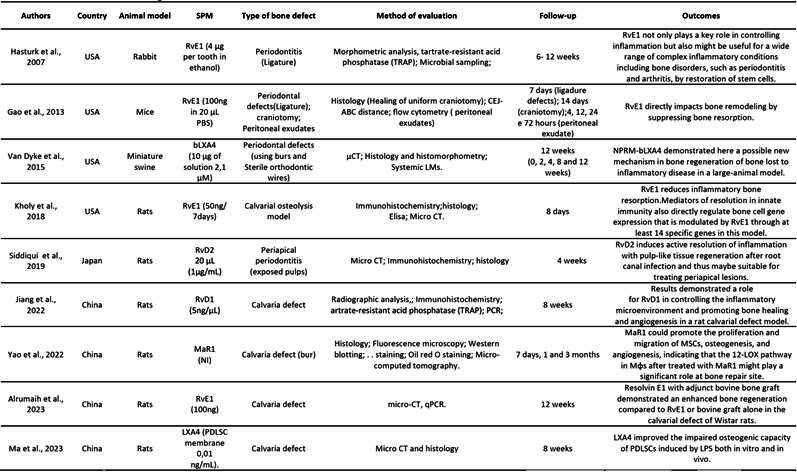
Characteristics of the studies using animal models

bLXA4 (benzo-lipoxin A4); CEJ-ABC ( distance between cementoenamel junction - alveolar bone crest); LM (proresolving lipid mediators); LPS (lipopoly-saccharide); LXA4 (Lipoxin A4); MaR1(Maresin-1); MNGCs (multinucleated giant cells);Mφs (host macrophages); NPRM (nano-proresolving medicines); PDLCSs (periodontal ligament stem cells; RvE1 (Resolvin E1); RvD1 (Resolvin D1); RvD2 (Resolvin D2); USA (United States of America); 12-LOX (12-lipoxygenase).

**Box 2:**
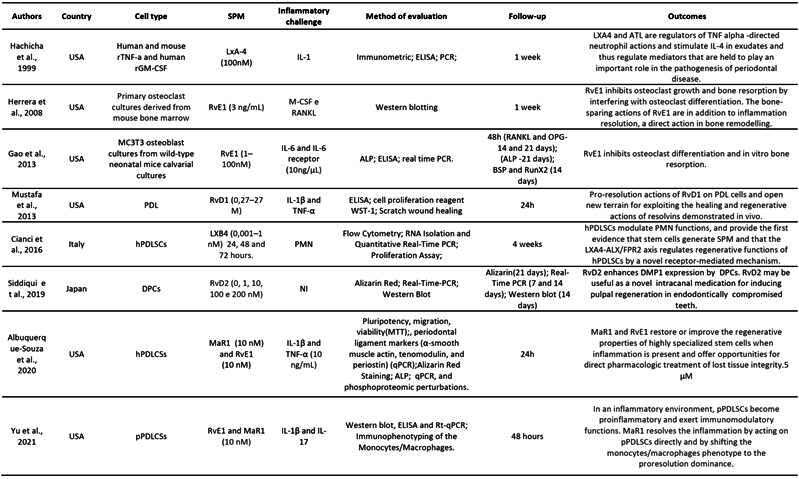
Characteristics of the in vitro studies

ALP(Alkaline phosphatase staining); ATL(aspirin-triggered-lipoxins);BSP (bone sialoprotein); cys-SPMs (cysteinyl-containing SPMs) ; DMP1 (dentin matrix acidic phosphoprotein 1); DPCs (primary dental pulp cells); FTIR (Fourier-transform infrared spectroscopy); hMSCs (human mesenchymal stem cells); hPDLCSs (human periodontal ligament stem cells); hBMMSCs (human bone marrow-derived mesenchymal stem cells); IL-17 (interleukin 17); IL-1β (interleukin 1β ); IL-6 (interleukin 6); TNF- α (tumor necrosis factor); LC/MS (liquid chromatography mass spectrometry); LPS (lipopoly-saccharide); LXA4 (Lipoxin A4); LXA4-ALX/FPR2 (specific G-protein-coupled receptor named) LXA4-ALX/FPR2; MaR1(Maresin-1); MCTR3 (maresin conjugates in tissue regeneration 3 ); mMSCs (Mouse mesenchymal stem cells); Mφs (host macrophages); NI (not informed); PDL (human periodontal ligament fibroblasts); PDLCSs (periodontal ligament stem cells); PMN (polymorphonuclear neutrophil); pPDLCSs (periodontal ligament stem cells of the Yorkshire pig); RvE1 (Resolvin E1); RvD1 (resolvin D1);SEM (Scanning electron microscopy); USA (United States of America); 12-LOX (12-

**Box 2:**
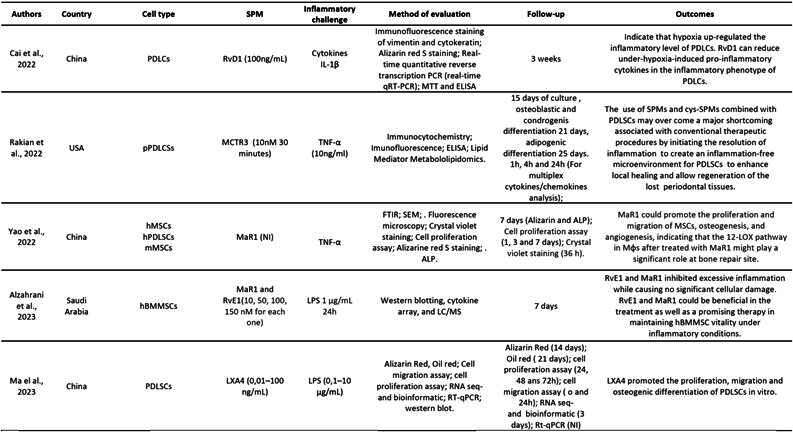
Continuation

Furthermore, LxA-4 and RvE1 in cell cultures from different origins, demonstrate the ability of these mediators to inhibit osteoclast differentiation and bone resorption, as well as to promote inflammation resolution ^5^ ^,^ ^8^ ^,^ ^9^. Tests with MaR1 and RvE1 in specialized stem cells showed that these SPMs can restore or improve the regenerative properties of these cells in inflammatory environments, in addition to inhibiting excessive inflammation ^11^ ^,^ ^12^ ^,^ ^14^.

Meta-analysis was performed with the data from 6 studies with animal models ^1^ ^,^ ^7^ ^,^ ^10^ ^,^ ^13^ ^,^ ^16^ ^,^ ^19^. Pooled estimates indicated significant differences between the SPMs and the control groups for the percentage of newly formed bone (%) (WMD 14.85; 95% CI: 12.07,17.62; p<0.00001), the area of the remaining defect (mm^2^) (WMD 0.35; 95% CI: 0.32,0.38; p<0.00001), and the linear distance from the defect to the bone crest (mm) (WMD 0.53; 95% CI: 0.51,0.54; p<0.00001) in animal models.

**Figure 3:**
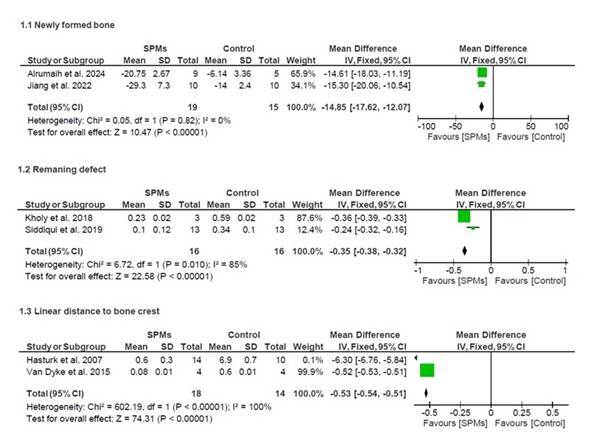
Meta-analysis assessing studies in animal models.

## Discussion

In an era characterized by rapid technological advancements and evolving scientific paradigms, the synergistic integration of manual and technological approaches, coupled with specialized expertise, becomes crucial to ensure the validity and integrity of the results of systematic reviews. Also due to rapid technological and scientific advances, systematic reviews are necessary to substantiate and round off all this knowledge produced.

The main results of this review showed that the use of SPMs can be considered a promising approach in alveolar bone and craniofacial bone defect repair. These results were based on pre-clinical studies, in vivo ^1^ ^,^ ^7^ ^,^ ^9^ ^,^ ^10^ ^,^ ^13^ ^,^ ^15^ ^,^ ^16^ ^,^ ^18^ ^,^ ^19^ and in vitro ^5^ ^,^ ^8^ ^,^ ^9^ ^,^ ^11^ ^,^ ^12^ ^,^ ^14^ ^,^ ^15^ ^,^ ^17^ ^,^ ^18^ ^,^ ^19^ ^,^ ^20^ ^,^ ^21^ ^,^ ^22^, and provide an overview of updated literature on the use of SPMs in the field of tissue engineering. The meta-analysis showed that SPMs can increase the percentage of newly formed bone by 14.8%, reduce the area of the remaining defect by 0.35mm^2^, and decrease the distance between the defect and the bone crest by 0.53mm in animal models.

Maresins, mainly MaR1 ^11^ ^,^ ^12^ ^,^ ^14^ ^,^ ^15^, and E-series resolvins, mainly RvE1 ^7^ ^,^ ^8^ ^,^ ^9^ ^,^ ^10^ ^,^ ^11^ ^,^ ^12^ ^,^ ^13^ ^,^ ^14^, have been the two most studied SPMs to date in the context of bone regeneration. There are also reports of the use of lipoxins (LxA4, LxB4, and bLXa4) ^5^ ^,^ ^16^ ^,^ ^17^ ^,^ ^18^, as well as D-series resolvins, including RvD1 ^1^ ^,^ ^20^ ^,^ ^21^ and RvD2 ^19^.

Regarding the animal models, experiments were conducted mostly in rats, with a calvarial defect model or induction of periodontitis ^1^ ^,^ ^7^ ^,^ ^9^ ^,^ ^16^ ^,^ ^10^ ^,^ ^14^ ^,^ ^15^ ^,^ ^18^. All the animal studies evaluated the use of SPMs in the context of bone tissue repair ^1^ ^,^ ^10^ ^,^ ^13^ ^,^ ^18^ and periodontitis treatment ^7^ ^,^ ^9^ ^,^ ^16^. The three included studies that replicate periodontitis models ^7^ ^,^ ^9^ ^,^ ^16^ used RvE1 (2 studies ^7^ ^,^ ^9^) and bLXA4 (1 study ^16^) respectively, and demonstrate the direct relationship between SPMs and osteoclastogenesis inhibition and bone formation. These results corroborate the findings of four in vitro studies ^8^ ^,^ ^9^ ^,^ ^15^ ^,^ ^18^ indicating a role of SPMs in the osteoclast differentiation inhibition, inflammation resolution, and bone remodeling of osteoclasts.

Considering the calvarial defect models ^1^ ^,^ ^10^ ^,^ ^13^ ^,^ ^15^ ^,^ ^18^, several SPMs were evaluated. In addition to RvE1 and bLXA4, there are reports of MaR1, RvD2, and RvD1 which also demonstrate their important role in bone healing and angiogenesis. The results are as favorable as those obtained in the periodontal disease models mentioned above and also correlate SPMs with osteogenesis.

Thirteen in vitro studies on the use of SPMs in bone repair were included in this systematic review ^5^ ^,^ ^8^ ^,^ ^9^ ^,^ ^11^ ^,^ ^12^ ^,^ ^14^ ^,^ ^15^ ^,^ ^17^ ^,^ ^18^ ^,^ ^19^ ^20^ ^,^ ^21^ ^,^ ^22^. Of these, three also show an additional in vivo experiment ^9^ ^,^ ^15^ ^,^ ^18^. The results found herein were similar to those in the abovementioned animal models. Studies evaluated different types of stem cells, showing a potential regenerative role of SPMs in bone cell differentiation, increased calcium deposit formation ^9^ ^,^ ^17^ ^,^ ^18^, regenerative and healing potential in scratch tests ^11^ ^,^ ^17^ ^,^ ^19^, decreased inflammatory activity exerted by pro-inflammatory cytokines (RANKL, TNF-α, and IFN-γ), and increase in anti-inflammatory cytokines (IL-10, IL-6, IL-4, CXCr-4, TGF- β1) in mesenchymal cells ^14^ ^,^ ^15^.

Moreover, SPMs can affect different inflammatory cells. MaR1 exerts immunomodulatory effects in periodontal ligament stem cells, which in turn can act on the monocytes/macrophages phenotype shifting to a pro-resolution dominance ^12^. Interestingly, one study evaluated a MaR1 scaffold and found an action on macrophage chemotaxis, which may promote greater polarization towards M2 macrophages (pro-resolution macrophages) to guide bone regeneration, in addition to positively regulating some anti-inflammatory cytokines (IL-6, IL-4, IL-10; TNF- α, CXCR-4) ^15^.

SPMs are essential in modulating the immune system by promoting control of neutrophil recruitment, facilitating phagocytosis of resident macrophages, and inducing cell apoptosis in inflamed sites. These biological mechanisms are caused by the negative regulation of pro-inflammatory cytokines and chemokines (TNF-α, IL-1β, and IL-6) and the promotion of the synthesis of anti-inflammatory cytokines (IL-10 and TGF-β) ^16^ ^,^ ^20^. This modulation is mediated through interaction with specific G protein-coupled receptors, ALX/FPR2, GPR32 and ChemR23, which are responsible for intracellular signaling cascades that act on pro-inflammatory genes and activation of resolution pathways ^16^ ^,^ ^19^ ^,^ ^23^. Additionally, SPMs increase efferocytosis, where macrophages engulf and eliminate apoptotic cells and cellular debris, a vital step for the orderly resolution of inflammation and allowing a better regenerative condition for the tissue involved ^7^ ^,^ ^23^.

Some limitations of the present scoping review was the methodological variances across studies, including the use of diverse SPMs, treatment regimens, animal models, and treated conditions, introduce significant heterogeneity. This is why this review was not registered in PROSPERO. Thus, standardizing future experimental protocols would enable smoother translational endeavors toward the clinical applications of SPMs in bone regeneration.

In summary, SPMs have emerged as pivotal contributors to the resolution of inflammation and the facilitation of bone neoformation within craniofacial defects. These results are based on pre-clinical studies, in vivo and in vitro, and provide an updated systematic review regarding the impact of SPMs in tissue engineering. The regenerative mechanisms of SPMs encompass the regulation of osteoclastogenesis, modulation of angiogenesis, and orchestration of immunomodulatory responses. While the potential of SPMs in ameliorating bone defects and addressing periodontal ailments appears promising, this review underscores the necessity for cautious interpretation.
